# Insecticide resistance in *Anopheles arabiensis* populations from Dakar and its suburbs: role of target site and metabolic resistance mechanisms

**DOI:** 10.1186/s12936-018-2269-6

**Published:** 2018-03-15

**Authors:** A. Kane Dia, O. Kalsom Guèye, E. Amadou Niang, S. Mocote Diédhiou, M. Demba Sy, Abdoulaye Konaté, Badara Samb, Abdoulaye Diop, Lassana Konaté, Ousmane Faye

**Affiliations:** 1 Laboratoire d’Ecologie Vectorielle et Parasitaire, Université Cheikh Anta Diop, Dakar, Senegal; 2 Aix Marseille Univ, IRD, APHM, MEPHI, IHU-Méditerranée Infection, Marseille, France; 3 Abt Associates, PMI Africa Indoor Residual Spraying Project, Dakar, Senegal

**Keywords:** *Anopheles arabiensis*, Dakar, L1014S, L1014F, Insecticide resistance, Metabolic, Synergist, WHO, CDC bottle

## Abstract

**Background:**

Urban malaria is an increasing concern in most of the sub-Saharan Africa countries. In Dakar, the capital city of Senegal, the malaria epidemiology has been complicated by recurrent flooding since 2005. The main vector control measure for malaria prevention in Dakar is the community use of long-lasting insecticide-treated nets. However, the increase of insecticide resistance reported in this area needs to be better understood for suitable resistance management. This study reports the situation of insecticide resistance and underlying mechanisms in *Anopheles arabiensis* populations from Dakar and its suburbs.

**Results:**

All the populations tested showed resistance to almost all insecticides except organophosphates families, which remain the only lethal molecules. Piperonil butoxide (PBO) and ethacrinic acid (EA) the two synergists used, have respectively and significantly restored the susceptibility to DDT and permethrin of *Anopheles* population. Molecular identification of specimens revealed the presence of *An. arabiensis* only. *Kdr* genotyping showed the presence of the L1014F mutation (*kdr*-West) as well as L1014S (*kdr*-East). This L1014S mutation was found at very high frequencies (89.53%) in almost all districts surveyed, and in association with the L1014F (10.24%).

**Conclusion:**

Results showed the contribution of both target-site and metabolic mechanisms in conferring pyrethroid resistance to *An. arabiensis* from the flooded areas of Dakar suburbs. These data, although preliminary, stress the need for close monitoring of the urban *An. arabiensis* populations for a suitable insecticide resistance management system to preserve core insecticide-based vector control tools in this flooded area.

## Background

Malaria is still a major public health emergency across the sub-Saharan Africa [1]. The Senegalese Malaria Control Programme has made unprecedented progresses against the disease, and is now targeting malaria pre-elimination/elimination in eligible areas. However, despite the encouraging results, malaria stills endemic in the most parts of the country, including Dakar and its suburbs where the epidemiology of the disease has been locally complicated by recurrent flooding since 2005. Indeed, floods have created suitable conditions for the persistence of *Anopheles arabiensis* larval habitats year-round [2–6]. In this context, the subsequent upsurge of vector populations’ densities increases the risk of malaria transmission in such a high-density of non-immune population. Moreover, the above-mentioned successes were made possible by scaling-up effective malaria control interventions, including the two core malaria vectors control tools: long-lasting insecticide-treated nets (LLIN) and indoor residual spraying (IRS). Indeed, across the sub-Saharan Africa, the proportion of the population at risk sleeping under an insecticide-treated net or protected by IRS increased from 37% in 2010 to 57% in 2015 [7].

In Senegal, as part of the malaria control effort with the U.S. President’s Malaria Initiative (PMI), the IRS programme has been introduced then scaled-up in different eco-epidemiological areas of the country [8, 9]. High reliance on these insecticide-based interventions has subjected the targeted vectors populations to an increasing insecticide pressure for the selection of the resistance phenotypes. The spread of the resistance to the main insecticide classes approved by the World Health Organization (WHO) for use in public health threatens the success of the pre-elimination and elimination programmes in Senegal [8]. In areas such as the western coastal areas, where the impact of climate change is most felt, the conjunction of climate hazards and insecticide resistance will increase the risk and the heterogeneity of malaria epidemiology [10]. Therefore, monitoring insecticide resistance in the main urban malaria vector, *An. arabiensis*, is essential for planning and implementing an effective vector control programme in this area.

This study was undertaken to characterize insecticide resistance and underlying mechanisms among the urban *An. arabiensis* populations across the flooded areas of the Dakar suburbs, the capital of Senegal.

## Methods

### Study area

**Fig. 1:**
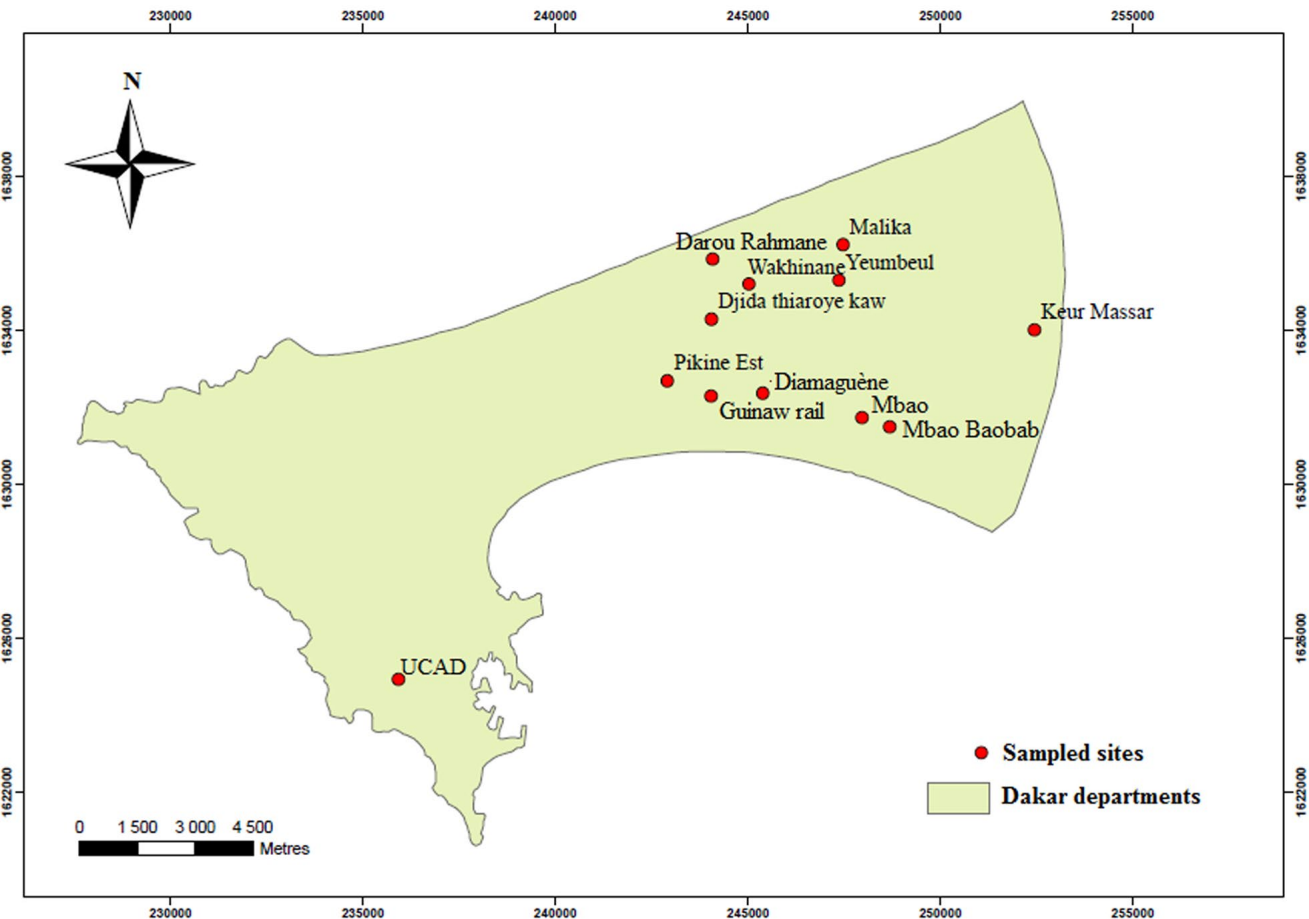
Study sites

The study was conducted during three successive years 2013, 2014 and 2015. Ten sites of Dakar and its suburbs (Pikine and Guediawaye) were surveyed during the 2013 rainy season and 2014 dry season for the first round of the study, then during the 2015 rainy season a second round was conducted only in the suburbs area of the administrative department of Pikine and Guediawaye (Fig. 1 and Table 1). The study area, located in a specific eco-geographical zone known as “Niayes”, is characterized by interdunal depressions, which are flooded during the rainy season. The region belongs to the sudano-sahelian domain with a rainy season from July to October and a dry season from November to June. The region average temperatures ranging from 24 to 30 °C during the rainy season and from 19 to 25 °C during the dry season. For the year 2013, 2014 and 2015 the annual rainfall was 567, 161 and 635 mm, respectively [11]. Dakar is the most populated Senegalese region with a density of 5404 inhabitants per km^2^ [12].

**Table 1:** Insecticide molecules and diagnostic concentrations used for each study locality and season

Districts	RS 2013	DS 2014	RS 2015	
	WHO papers	WHO papers	WHO papers	CDC-bottle
Dakar centre	Bendiocarb 0.1% Pirimiphos methyl 0.25%	–	–	–
Mbao	DDT 4% Deltamethrine 0.05% Alphacypermethrin 0.05% Bendiocarb 0.1% Pirimiphos methyl 0.25%	DDT 4% Permethrin 0.75% Deltamethrin 0.05% Bendiocarb 0.1% Malathion 5% Pirimiphos methyl 0.25%	–	–
Pikine	Dieldrin 4% Permethrin 0.75%	–	DDT 4% Dieldrin 4% Permethrin 0.75% Deltamethrin 0.05% Alphacypermethrin 0.05% Cyfluthrin 0.15% Lambda-cyhalothrin 0.05% Bendiocarb 0.1% Malathion 5% Pirimiphos methyl 0.25% Fenitrothion 1%	DDT 4% Permethrin 0.75% Deltamethrin 0.05% Bendiocarb 0.1% Pirimiphos methyl 0.25%
Keur Massar	DDT 4% Bendiocarb 0.1% Malathion 5% Fenitrothion 1%	DDT 4% Permethrin 0.75% Deltamethrin 0.05% Bendiocarb 0.1% Malathion 5% Pirimiphos methyl 0.25%	–	–
Guediawaye	Deltamethrin 0.05%	–	DDT 4% Dieldrin 4% Permethrin 0.75% Deltamethrin 0.05% Alphacypermethrin 0.05% Cyfluthrin 0.15% Lambda-cyhalothrin 0.05% Bendiocarb 0.1% Malathion 5% Pirimiphos methyl 0.25% Fenitrothion 1%	DDT 4% Permethrin 0.75% Deltamethrin 0.05% Bendiocarb 0.1% Pirimiphos methyl 0.25%

### Sampling and rearing mosquito larvae

*Anopheles* larvae and pupae were collected from natural breeding sites within and around the study sites. Upon collection, they were kept in separate labelled buckets, transported to the insectary and maintained under optimal rearing condition at a relative humidity of 75 ± 5% and a temperature of 28 ± 2 °C as described in the MR4 manual for mosquitoes rearing [13].

### Insecticide susceptibility tests

In 2013 and 2014, bioassays were carried out using only the WHO test kits for adult mosquitoes [14]. While, for the 2015 study year, populations were tested using both the WHO standard test and the CDC Bottle test [14, 15]. The WHO insecticide-impregnated papers were provided by The Vector Control Research Unit, School of Biological Sciences (Universiti Sains Malaysia), a WHO Collaborating Centre, while insecticide for the bottle test were provided by the Centers for Disease Control and Prevention (CDC), Atlanta, USA.

Non-blood-fed, 3–5 days old adults females of *Anopheles gambiae* sensu lato (s.l.) were exposed to diagnostic concentrations of insecticides for the required diagnostic time for each molecule [14]. For each insecticide, four tests and two control replicates of 25 mosquitoes were used for each test round.

### Detection of metabolic resistance mechanisms

To assess the presence of the metabolic resistance mechanisms, 3–5 days old of non-blood-fed *An. gambiae* s.l. adult females were pre-exposed to the piperonyl butoxide (PBO) or ethacrynic acid (EA) prior to be exposed to insecticides [15].

### Species identification and kdr molecular genotyping

Genomic DNA was extracted from individual mosquitoes as described by CTAB method and the member of the *An. gambiae* complex were identified as described by Wilkins et al. [16]. The *kdr* mutations molecular genotyping was performed as described in Huynh et al. [17].

### Data analysis

WHO susceptibility and CDC bottle tests were used to monitor resistance to insecticides belonging to the four WHO-approved chemical classes over the study period. The *kdr* allele frequency was estimated for each site and period of collection as the proportion of specimens with the L1014F and/or L1014S *kdr* alleles. All statistical analyses were performed using R soft-ware (version 3.3.2) [18].

## Results

### Species identification and susceptibility to insecticide

Molecular identification revealed the exclusive presence of *An. arabiensis* in all the study areas and sites. WHO susceptibility tests were performed on 1414; 1687 and 3316 *An. arabiensis* specimens, respectively in 2013, 2014 and 2015 (Table 1). *Anopheles arabiensis* populations were resistant to almost all insecticides, but generally fully susceptible to organophosphates.

In 2014, the studied population displayed pronounced resistance to pyrethroids and DDT, especially in Mbao during the rainy season where populations were fully resistant to the permethrin. Excepted for the dieldrin (organochlorine) and the cyfluthrin (pyrethroid) in Pikine, both *An. arabiensis* populations in Guediawaye and Pikine were resistant to DDT and pyrethroids during the 2015 rainy season (Table 2).

**Table 2:** Twenty-four hours post-exposure mortality (%) to pyrethroid (PYR) and organochlorine (OC) using the WHO impregnated papers

	Districts	Localities	OC		PYR				
			DDT	Dieldrin	Permethrin	Deltamethrin	Alpha cypermethrin	Cyfluthrin	Lambda cyhalothrin
RS 2013	Guediawaye	Wakhinane	–	–	–	65 (100)	–	–	–
	Keur Massar	Keur Massar	4 (91)	–	–	–	–	–	–
	Mbao	Mbao Baobab	7 (95)	–	–	73 (94)	–	–	–
		Diamaguene	–	–	–	–	38 (98)	–	–
	Pikine	Pikine Est	–	14 (93)	39 (93)	–	–	–	–
DS 2014	Keur Massar	Yeumbeul	1 (85)	–	2 (102)	69 (97)	–	–	–
	Mbao	Mbao	3 (103)	–	0 (94)	46 (102)	–	–	–
RS 2015	Guediawaye	Darou Rahmane	0.98 (102)	31 (124)	3 (102)	47 (104)	42.59 (108)	31.69 (124)	17 (112)
	Pikine	Guinaw Rails	1 (113)	14 (103)	21.81 (110)	62 (110)	64. 22 (123)	25.04 (104)	23 (100)

*RS* rainy season, *DS* dry season

() Numbers between brackets indicate the total of specimens tested

All *An. arabiensis* populations were resistant to the bendiocarb during the whole study period except in Yeumbeul (98.81%) in 2013. Excepted Mbao Baobab and Pikine, where suspected resistance to Malathion was noticed in 2014; and for Guediawaye populations which were resistant to Fenitrothion during the 2015 rainy season, all the studied populations were fully susceptible to organophosphates (Table 3).

**Table 3:** Twenty-four hours post-exposure mortality (%) to carbamate (CAR) and organophosphate (OP) using the WHO impregnated papers

	Districts	Localities	CAR	OP		
			Bendiocarb	Pyrimiphos methyl	Malathion	Fenitrothion
RS 2013	Dakar centre	UCAD	2.99 (93)^a^	100 (87)	–	–
	Keur Massar	Yeumbeul	98.81 (93)	–	–	–
		Keur Massar	–	–	100 (96)	–
		Malika	–	–	–	100 (101)
	Mbao	Mbao Baobab	88 (86)	100 (97)	–	–
DS 2014	Keur Massar	Yeumbeul	27.03 (74)	100 (86)	100 (99)	–
	Mbao	Mbao	0.96 (104)	–	–	–
		Mbao Baobab	–	–	94 (98)	–
		Djida Thiaroye	–	100 (91)	–	–
RS 2015	Guediawaye	Darou Rahmane	35.17 (107)	100 (103)	100 (102)	73. 05 (106)
	Pikine	Guinaw Rails	62 (120)	100 (118)	95 (120)	100 (103)

*RS* rainy season; *DS* dry season

() Numbers between brackets indicate the total of specimens tested

^a^Corrected mortality corrected

In 2015 the susceptibility tests were carried out for the permethrin, deltamethrin, DDT, pirimiphos-methyl and bendiocarb using the standard WHO-impregnated paper test as well as the CDC Bottle test. Both methods have shown that *An. arabiensis* populations from Guediawaye and Pikine were resistant to DDT and pyrethroids (Tables 2, 3, 4). However, the results were different for the two remaining molecules; with both populations being fully susceptible to the pirimiphos-methyl but resistant to the bendiocarb using the impregnated paper test; while an opposite trend was recorded for the Bottle test.

**Table 4:** Post-exposure mortality (%) to pyrethroid (PYR), organochlorine (OC), carbamate (CAR) and organophosphate (OP) using the CDC bottles test

Districts	Localities	OC	PYR		CAR	OP
		DDT	Permethrin	Deltamethrin	Bendiocarb	Pirimiphos-methyl
Guediawaye	Darou Rahmane	1.98 (101)	86.66 (105)	93.45 (107)	100 (96)	61.22 (98)
Pikine	Guinaw Rails	36.19 (105)	87.12 (101)	83.01 (106)	100 (106)	55.66 (106)

### Resistance mechanisms

**Fig. 2:**
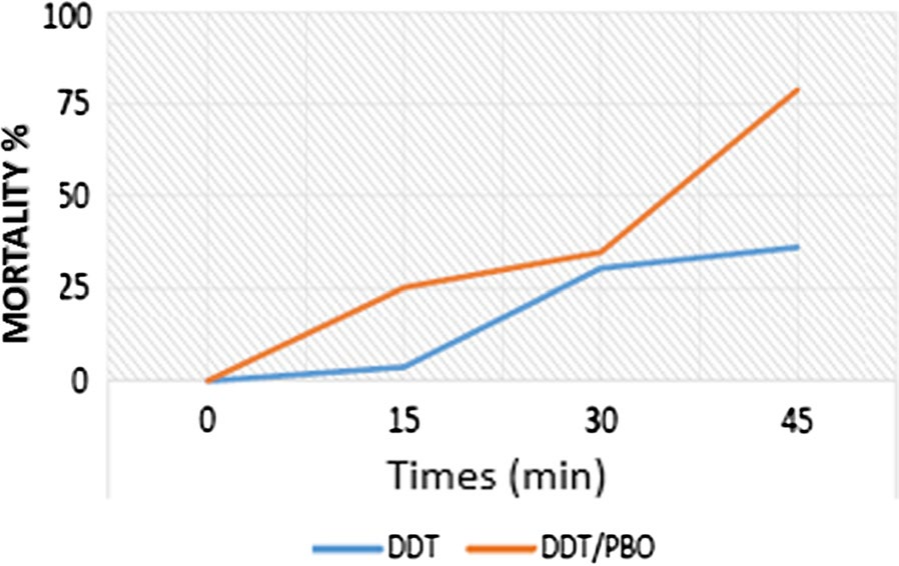
Partial restauration of the susceptibility to DDT in resistant populations of *Anopheles arabiensis* after pre-exposure to PBO

**Fig. 3:**
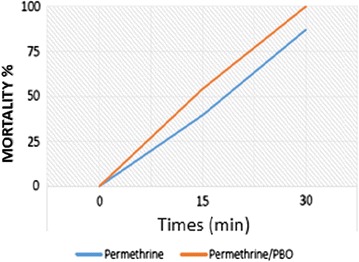
Partial restauration of the susceptibility to permethrin in resistant populations of *Anopheles arabiensis* after pre-exposure to PBO

**Fig. 4:**
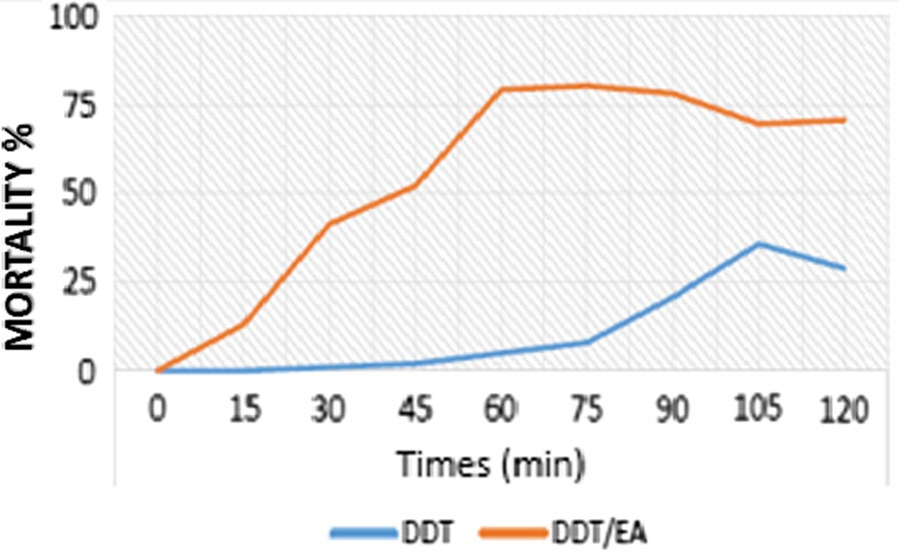
Partial restauration of the susceptibility to DDT in resistant populations of *Anopheles arabiensis* after pre-exposure to EA

The CDC Bottle test with synergists was used to assess the presence of the metabolic resistance mechanisms. Results indicate the involvement of metabolic resistance mechanisms via *GST* and *CYP450* detoxification genes families. In Pikine, pre-exposure to PBO has significantly restored the susceptibility of tested *An. arabiensis* populations to DDT with increased mortality from 36.19 to 78.95%; while complete restoration of susceptibility to permethrin was noted (from 87.12 to 100%) (Figs. 2 and 3). A 25-fold (1.98 to 52.17%) restoration of susceptibility to DDT was recorded for Guediawaye populations after pre-exposure to EA suggesting a GST-mediated metabolic resistance (Fig. 4).

**Fig. 5:**
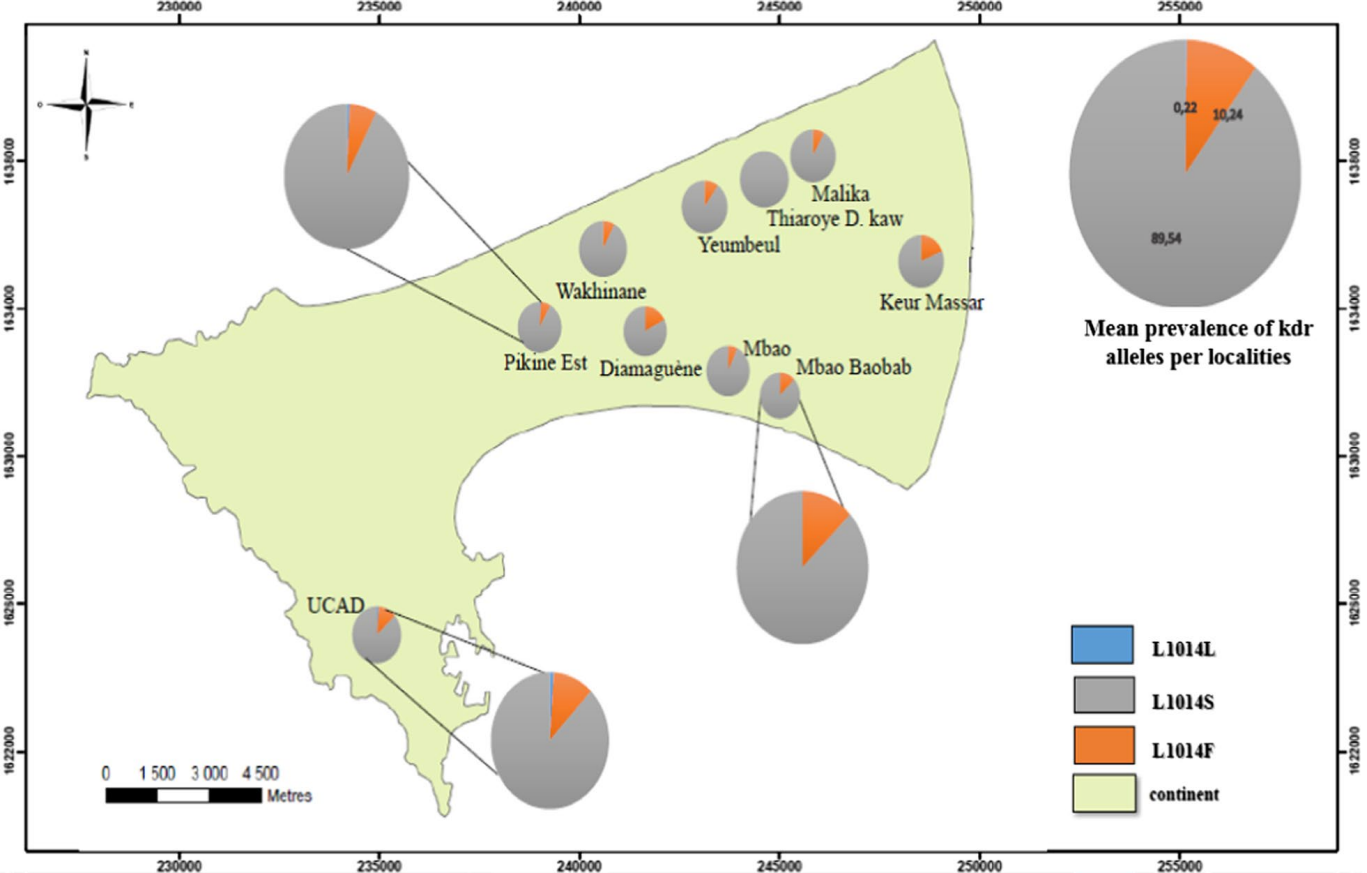
Distribution of the 1014L, 1014F and L1014S alleles frequencies in Dakar in 2013 and 2014

The *kdr* (L1014F and L1014S) mutations, conferring cross-resistance to DDT and pyrethroids, have been also investigated. Except in Djida Thiaroye Kaw where only the L1014S mutations was found, both mutations were present in all the study sites with respective frequencies of 10.24 and 89.53% for the West African (L1014F) and East African (L1014S) mutations. Notably, the East African mutations was significantly higher than the West African one (p < 0.05). Both mutations were identified either at homozygous or heterozygous resistant genotype varying between study sites. The homozygous L1014S resistant genotype was the most common genotype in all the surveyed sites with the highest frequency recorded in Mbao, while the less common genotype was the heterozygous 1014S/1014L with the lowest accounted only for 1.56% of all the genotype in Pikine (Fig. 5).

## Discussion

During this study, *An. arabiensis* was the sole member of the Gambiae complex encountered in all the surveyed sites. This confirms this species as the unique or main representant of the Gambiae complex in Dakar [2, 5, 6, 19]. No specimen of *Anopheles melas* or *Anopheles coluzzii*, previously reported [6, 20] was found over the study period. During their study Gadiaga et al. [6] collected *An. melas* both at the larval and adult stages. *Anopheles melas* larvae were often collected in association with *An. arabiensis *from a variate breeding sites located in Cafeteriat, Pikine and Zone A, in Dakar suburb. The absence of *An. melas* from the larval collection may be explained by the lower sampling effort compared to the previous study. A recent study carried-out in the same geographical area reported also only the presence of *An. arabiensis* in both larval and adult anopheline populations [2, 3].

Historically, *An. arabiensis* is considered as the main malaria vector in the Cap Vert Peninsula where it is found all the year-round [21, 22]. Robert et al. [21] attributed this to the presence of permanent larval habitats formed by the so-called “Ceane” gardening pits which served as breeding site, especially during the dry season. Gadiaga et al. [6] found significant difference in larval habitats conductivity between breeding sites containing both *An. melas* and *An. arabiensis* (5.03) and those exclusive for *An. arabiensis* (1.75), with the conductivity of the water being the highest when *An. melas* was present.

WHO susceptibility tests showed that *An. arabiensis* populations were resistant to three classes of insecticides, but susceptible to organophosphates. The pronounced resistance to pyrethroids in the study populations is consistent with the overall situation reported for malaria vectors to this chemical family across the sub-Saharan Africa [1].

The study populations were more susceptible to organophosphate compared to carbamate. Indeed, all *An. arabiensis* populations were resistant to the bendiocarb except in Yeumbeul during the 2013 rainy season. Similar results were previously reported in the country including the study area [23]. This situation may be explained by an extensive use of insecticide for crops protection in the market gardening activities in the Niayes area [24, 25]. Moreover, Faye et al. [26] have previously reported a resistance of *An. gambiae* s.l. to DDT in the Niayes. The current cross-resistance of *An. arabiensis* populations to pyrethroids and DDT may be an heritage of an extensive agricultural used of DDT in the past as hypothesized in Burkina Faso [27]. However, Padonou et al. [28] attributed it rather to the use of pyrethroids in public health.

Both the L1014F (*kdr*-*West* or *kdr*-*w*) and the L1014S (*kdr*-*East* or *kdr*-*e*) mutations, the two target site mechanisms conferring a cross-resistance to DDT and pyrethroids were found in almost all the study sites. Previous studies have reported the presence of the L1014F mutation nationwide with variable frequencies across the country and between studies [10, 25]. Indeed, the *kdr*-*w* mutation was previously reported in the study area by Pagès et al. [20]; and, as shown here, its frequencies have increased since this first description. During this study, the *kdr*-*e* mutation also found in study populations, was the most widespread and the most frequent *kdr* allele. Therefore, more investigations are necessary to assess the contribution of each mutations to the resistance level of *An. arabiensis* across its distribution range. Soderlund and Knipple [29] have reported that the *kdr*-*w* mutation confers a highest resistance level, while the *kdr*-*e* gives a selective advantage to the individual that carries it [30].

The ~ 20% co-occurrence of the two mutations in *An. arabiensis* as observed here is similar to previous observations from several other sub-Saharan African countries, including Burkina Faso [31], Tanzania [32], Cameroon [33, 34], Gabon [35] and Uganda [36].

The absence of organophosphates-carbamates cross-resistance suggests the absence of the *ace*-*1*^R^ mutation gene [37], which was not investigated during this study. However, there is an urgent need to investigate all potential insecticide resistance mechanisms for a suitable insecticide management system.

During this study, a proportion of mosquitoes did not harbour the *kdr* alleles while the populations were fully resistant, suggesting the existence of other resistance mechanisms. Further analysis revealed the involvement of metabolic resistance mechanisms implying GST and *CYP450* detoxification genes families. Indeed, the susceptibility of studied populations to DDT and permethrin was fully restored following a pre-exposure to the PBO or EA. Similar results have been previously reported in different places in Dakar, including Fass and Colobane [38], in Benin [39, 40] and in Cameroon [41]. However, data presented in both studies are limited and need to be completed and updated to fully characterize the resistance of *An. arabiensis* populations to insecticide and underlying mechanisms.

## Conclusions

This study summarizes the insecticide resistance status and underlying mechanisms in *An. arabiensis* populations in the flooded areas of the Dakar suburbs. Noteworthily, it revealed the contribution of both target-site and metabolic resistance mechanisms in the study populations. These data, although preliminary, stress the need for close monitoring of the urban *An. arabiensis* populations for the implementation of a suitable insecticide resistance management system to preserve core insecticide-based vector control tools in this flooded area.
